# Immune metabolism regulation of the germinal center response

**DOI:** 10.1038/s12276-020-0392-2

**Published:** 2020-03-04

**Authors:** Seung-Chul Choi, Laurence Morel

**Affiliations:** Department of Pathology, Immunology, and Laboratory Medicine, University of Florida, Gainesville, FL USA

**Keywords:** Autoimmunity, Immunotherapy

## Abstract

The humoral immune response requires germinal centers to produce high-affinity antigen-specific antibodies that counter pathogens. Numerous studies have provided a better understanding of how metabolic pathways regulate the development, activation and functions of immune cells. Germinal centers are transient, highly dynamic microanatomic structures that develop in lymphoid organs during a T-cell-dependent humoral immune response. Analysis of germinal centers provides an opportunity to understand how metabolic programs control the differentiation and function of highly specialized germinal center B cells and follicular helper CD4^+^ T cells. Targeting immunometabolism during the germinal center response may afford the possibility to improve vaccine design and to develop new therapies to alleviate autoimmunity. In this review, we discuss the major metabolic pathways that are used by germinal center B and T cells, as well as the plasma cells that they produce, all of which are influenced by the microenvironment of this unique structure of the adaptive immune system.

## Adaptive immunity: Metabolic pathways need further investigation

Studies of the metabolic mechanisms involved in antibody production will inform vaccine design and autoimmune disease treatments. Germinal centers (GCs) are transient sites in lymph nodes and the spleen, formed when white blood cells called T-cell lymphocytes respond to infection. GCs act as factories where another lymphocyte group, B cells, proliferates and mutates before producing infection-appropriate antibodies. GCs therefore play a critical role in adaptive immunity, but the metabolic pathways involved are unclear. Laurence Morel and Seung-Chui Choi at the University of Florida, Gainesville, USA, reviewed understanding of the metabolic pathways used by T cells, B cells and the antibodies they produce. The cells within GCs require different energy sources and metabolic pathways according to their developmental stage, to ensure optimal immune responses. The researchers call for extensive profiling of this complex metabolic system.

## Introduction

Germinal centers (GC) are dynamic anatomical structures that provide a unique microenvironment for B-cell affinity maturation through somatic hypermutations (SHM) and class-switching recombination (CSR) to occur. A recent study using adoptive transfer of B-cell receptor (BCR) transgenic B cells challenged however this notion by showing that CSR predominantly takes place before GC formation^1^. Follicular helper (T_FH_) CD4^+^ T cells play an integral role in the formation of GC, as well as in the maturation and development of GC B cells into plasma cells (PCs) and memory B cells^2,3^. The GC is formed by two histologically distinct regions: the dark zone (DZ) consists of highly proliferative GC B cells in which CSR and SHM occur, and the light zone (LZ) where non-dividing GC B cells bind to cognate antigens presented as immune complexes by follicular dendritic cells (FDCs), to be selected through competition for stimulation by T_FH_ cells that are cognate for the same antigen^3,4^. This process promotes further clonal diversification through SHM after re-entry into the DZ, which results into the enhancement of antibody affinity^3^. It has been recently recognized that cells in the GC microenvironment have to cope with low amounts of oxygen, and that LZ B cells are hypoxic^5,6^. Hypoxia regulates immune functions at least partially through the alteration of nutrient utilization^7^. This prompted an examination of known or potential metabolic features of the GC response induced by pathogens or autoantigens. In addition, the different metabolic demands reported between autoreactive T_FH_ cells and pathogen-specific T_FH_ cells^8^ open a new window of opportunity to selectively target autoreactive cells and to dissect the mechanisms that distinguish the differentiation of pathogen- and autoantigen-induced T_FH_ cells^9^. We also review what is known about the metabolic regulation of follicular regulatory T (T_FR_) cells, a specialized subset of regulatory T (Treg) cells that exert immunosuppressive functions in GC^10^. Finally, recent studies have revealed complex metabolic regulation of B-cell differentiation that appears to be highly context and/or stage-specific, including long-lived plasma cells (LLPCs) that are derived from GC responses^11^. This review summarizes the key metabolic features of immune cells participating GC response, and discusses insights and future directions in light of recent studies.

### GC B cells

**Fig. 1:**
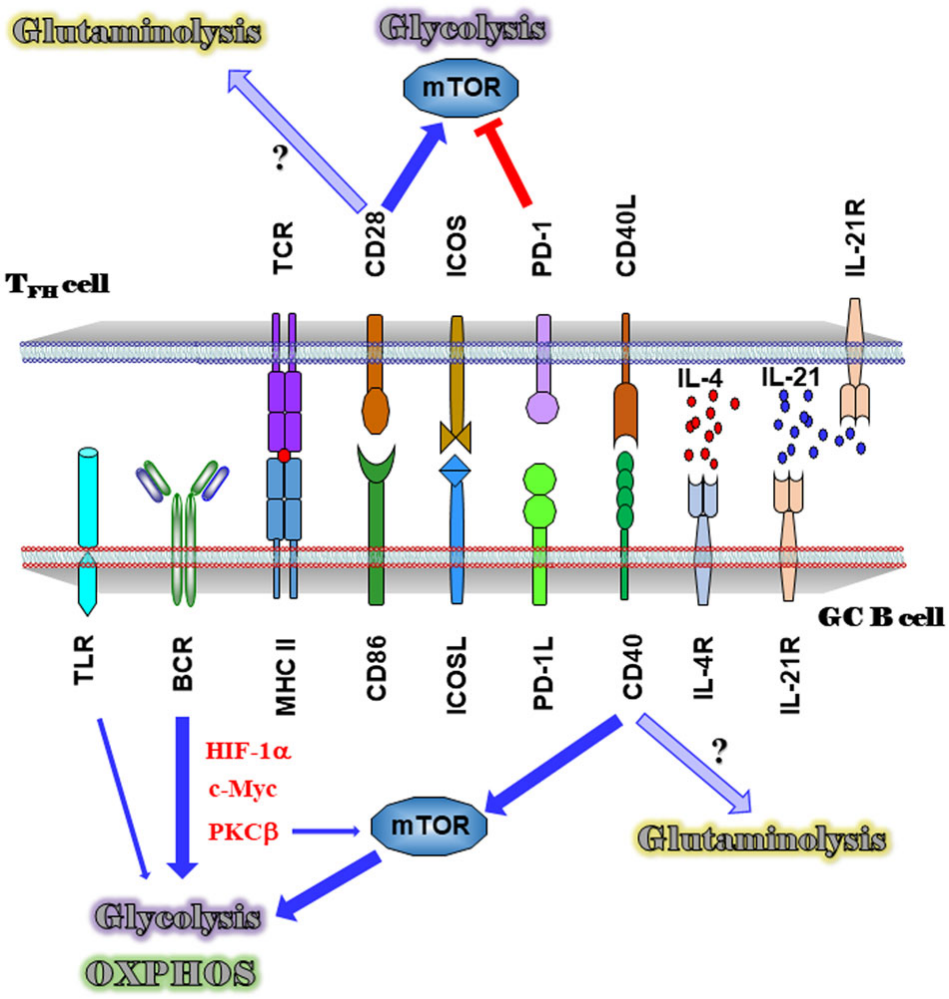
Metabolic consequences of the cross-talk between GC B cells and T_FH_ cells. BCR- or TLR-mediated activation induces glycolysis and OXPHOS in GC B cells via HIF-1α, c-Myc, and PKCβ. CD40 signaling is necessary to activate mTOR following this increased glycolysis and OXPHOS for GC B-cell survival and proliferation. Engagement of PD-1 expressed on T_FH_ cells with PD-1L expressed on GC B cells independently inhibits glycolysis, whereas CD28 signaling increases glycolysis in T_FH_ cells. Glutaminolysis is required by GC B cells and T_FH_ cells. Its potential activation by CD28 signaling in T_FH_ cells and CD40 signaling in GC B cells needs to be investigated.

B cells show increased glycolysis and oxidative phosphorylation (OXPHOS) after activation by a range of stimuli, and hypoxia-induced transcription factor HIF-1α and c-Myc directly bind to the promoters of genes encoding for glycolytic enzymes and glucose transporters^12–14^ (Fig. 1). Upon antigen recognition through a cognate B-cell receptor (BCR), B cells enter into GC, where they undergo changes that lead to the production of higher affinity antibodies. These responses are energetically demanding as previous reports showed that GC B cells increased glucose consumption and mitochondrial mass in comparison to those of naive B cells^6,15,16^. Indeed, the β isoform of protein kinase C (PKCβ), which is highly abundant in B cells and mediates proliferative signaling downstream of the BCR, is required for BCR-induced glycolysis^17^. Loss of PKCβ in B cells reduces the activation of the energy-regulating kinase complex mTORC1, resulting in a defective metabolism and mitochondrial remodeling, which leads to defective GC formation and generation of PCs^18^. Interestingly, mTORC1 is not required for the regulation of glycolysis in BCR-stimulated B cells^13^, whereas a pronounced increase of mTORC1 activity is observed in TCR-stimulated T cells^19^. However, mTORC1 signaling is necessary to positively select GC LZ B cells in a CD40-dependent manner, which leads to increased cell size, phosphorylation of S6 ribosomal protein, and migration to the GC DZ^15^. In addition, B cells lacking the GTPase R-Ras2, downstream of both the BCR and the costimulatory protein CD40, failed to proliferate and to be recruited to the GC response^20^. Loss of R-Ras2 in B cells inhibits the activation of the PI3K-Akt-mTORC1 pathway, reduces the replication of mitochondrial DNA, and decreases the expression of genes required for glucose metabolism.

**Fig. 2:**
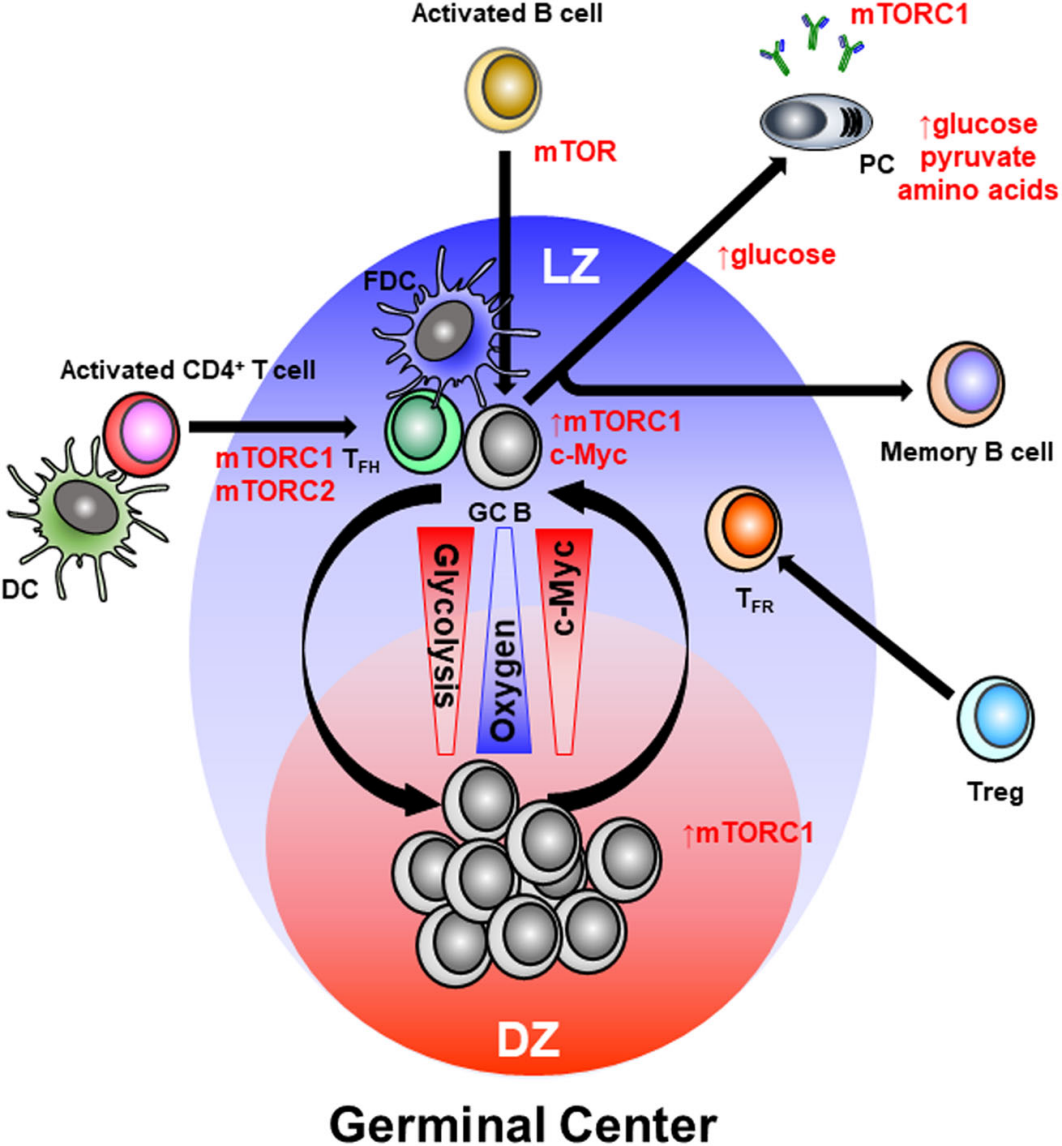
Overview of the major metabolic regulators in the GC. Activated B and CD4^+^ T cells migrate into the GC to differentiate GC B and T_FH_ cells through mTOR activation. LZ GC B cells are more glycolytic and show higher c-Myc expression than DZ GC B cells, which is correlated with their lower oxygen consumption in the LZ hypoxic environment. PCs continuously utilize glucose, which is the main energy source during B-cell activation, as well as pyruvate and amino acids.

Combined signals from BCR and CD40 in GC B cells induce c-Myc, a transcription factor regulating many cellular programs, including cellular metabolism and mTORC1 activation (Fig. 2). These two steps are necessary for the positive selection of GC B cells in the LZ, and their migration to the DZ, where rapid proliferation is metabolically demanding^21^. An elegant dissection of BCR signaling in GC B cells has identified a negative feedback in which AKT activates negative regulators of the proximal BCR signaling instead of, or in addition to, the downstream signals toward mTORC1 activation^22^. A new model suggests a complex balance between activating and inhibitory signals coupled with metabolic programming that controls GC B-cell selection and differentiation^23^. CD40 signaling is induced in GC B cells through their interaction with T_FH_ cells. A recent elegant study using c-Myc reporter mice has shown that this signal induces c-Myc expression by LZ GC B cells in direct proportion to the amount of antigen. Once GC B cells move to the DZ, c-Myc acts as the division timer by regulating DZ GC B-cell size and cell cycle entry^24^. As c-Myc levels get diluted at each cell division, GC B cells need to move back to the LZ for cognate Ag stimulation to start another cycle of selection.

A dynamic balance between metabolic activation and inhibition also occurs in GC B cells in response to oxygen sensing. In the LZ, low-oxygen tension promotes a high-glycolytic rate, which increases B-cell apoptosis, diminishes proliferation and impairs immunoglobulin class switching to the pro-inflammatory IgG2c isotype by limiting AID expression^5^. These features are required for the antigen-driven selection process in the GC LZ. HIF-1α expression is higher in GC B cells compared to that of other splenic B cells. Sustained hypoxia or HIF stabilization inhibits mTORC1 activity in B lymphoblasts in the DZ, which impairs their proliferation and class-switching^5^. This demonstrates that oxygen sensing and rapid switching to the corresponding metabolic program is an essential requirement of GC B cells.

**Fig. 3:**
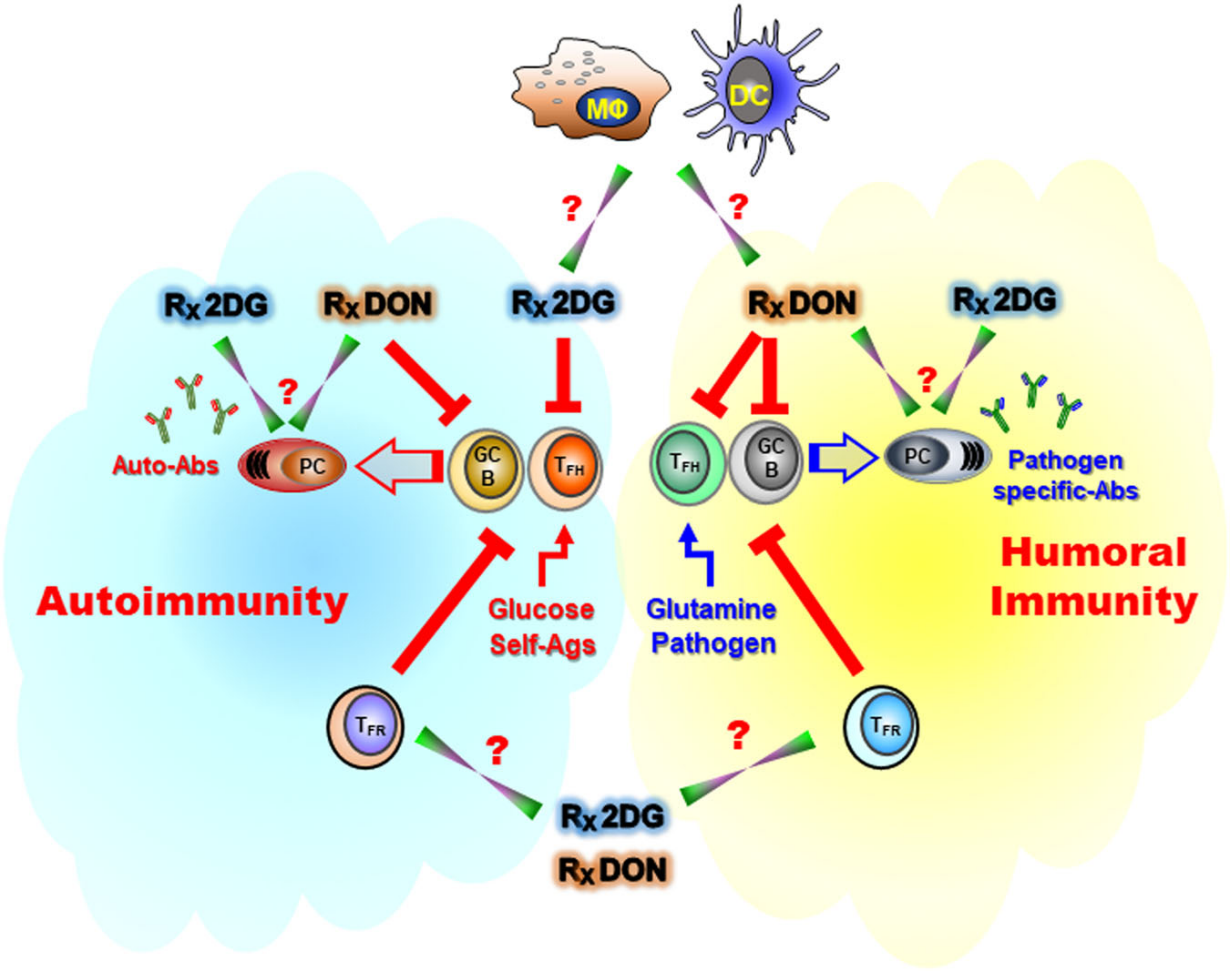
Proposed model of the requirements of GC B cells and T_FH_ cells for glucose and glutamine in response to autoimmune activation (left) or immunization with a foreign antigen (right). The production of class-switched antibodies, either in response to TD-antigens or autoantigens, requires glutamine and is blocked with DON. In contrast, only the spontaneous differentiation and expansion of T_FH_ cells in lupus-prone mice depends on glucose metabolism. This process and the subsequent GC B-cell expansion and autoantibody production is blocked with 2DG. On the other hand, exogenous Ag or pathogen-driven T_FH_ differentiation and expansion is glucose-independent, and therefore not affected by 2DG. The consequences of inhibiting glycolysis or glutaminolysis have not been examined in T_FR_ cells, PCs, FDCs, and tingible body macrophages. The labels above the cells show the effects of 2DG and DON. Red T lines indicate inhibition and green inverted triangles indicate cellular targets for which the effect has not yet been determined.

Treatment of normal or B6.*Sle1.Sle2.Sle3* lupus-prone mice using the hexokinase inhibitor, 2-deoxy-d-glucose (2DG), had no effect on the induction of antigen-induced GC B cells and corresponding antibodies, but it greatly reduced the induction of autoreactive GC B cells in lupus-prone mice^8^. It is not clear whether this difference corresponds to an intrinsic glucose requirement of autoreactive GC B cells, or if it corresponds to the differential glucose requirements of autoreactive and antigen-induced T_FH_ cells (see below). The fact that mTORC1 is not required for the regulation of glycolysis in BCR-stimulated B cells^13^ is consistent with antigen-induced GC B cells not being dependent on glycolysis. It is possible that the TLR7/TLR9 pathway, which plays a major role in the stimulation of autoreactive B cells^25,26^, is more glycolytic, explaining the glucose-dependency of autoreactive GC B cells. It is also possible that the nature of BCR stimulation (acute in immunization vs. chronic in autoimmunity) may determine the glucose requirements of GC B cells. Finally, the inhibition of glutaminolysis with DON (6-diazo-5-oxo-l-norleucine) greatly reduced immunization-induced as well as autoimmune humoral responses, in both lupus-prone and non-autoimmune mice, indicating that glutamine is required for GC development^8^ (Fig. 3). DON treatment greatly reduced the size of GC, and virtually eliminated GC B cells, although it had comparatively little effect on follicular B cells. The relative contribution of glucose and glutamine metabolism needs to be examined in details in both LZ and DZ GC B cells in both antigen-induced and spontaneous models. Furthermore, the contribution of BCR and TLR signaling, as well as T_FH_ cell co-stimulation (starting with CD40 signaling), needs to be dissected for a better understanding of the metabolic regulation of GC B cells.

### T_FH_ cells

T_FH_ cells are CD4^+^ helper T cells specialized in providing “help” to GC B cells in the form of co-stimulation through receptor/ligand pairs such as CD154/CD40 and cytokines such as interleukin (IL)-4 and IL-21. This help is essential in GC formation, affinity maturation, and the development of most high-affinity antibodies and memory B cells^27^. Upon TCR activation by cognate antigen on antigen-presenting DCs, naive T cells differentiate into pre-T_FH_ cells in the T-cell zone of secondary lymph organs. Pre-T_FH_ cells then migrate toward B-cell follicles where the subsequent GC reaction develops^28^ (Fig. 2). TCR-activated T cells undergo metabolic reprogramming toward glycolysis^29^, however, the subsequent step in T_FH_ cell differentiation is more reliant on mitochondrial oxidation^30–32^.

Bcl6^33^, the master regulator of T_FH_ cell gene expression, and PD-1^34^, which is highly expressed by T_FH_ cells, independently inhibit cellular metabolism, including glycolysis in vitro (Fig. 1). As IL-2 signaling through CD25 activates the PI3K-Akt-mTORC1 axis to promote glycolysis, IL-2-induced mTORC1 activity is necessary for induction of T_H_1 cell program but not for T_FH_ cell differentiation in the context of LCMV infection^31^. However, T-cell-specific genetic ablation of Raptor, a regulatory protein of the mTORC1 complex, decreases the frequency of T_FH_ cells^35^. Both mTORC1 and mTORC2 signaling are required for T_FH_ cell generation and optimal GC reaction, and overexpression of Glut1, the glucose transporter up-regulated upon T-cell receptor and co-stimulator CD28 signaling^30^, enhances T_FH_ cell differentiation and leads to autoimmunity^35,36^. Moreover, mTORC1 activation has been linked to the expansion of autoreactive T_FH_ cells by promoting the translation of Bcl6 in the *Def6*^*tr/tr*^*Swap70*^*−/−*^ DKO mice^37^. This nuanced mode of metabolic reprogramming may be due to an adaptation of T_FH_ cell differentiation to a unique niche of nutrient consumption that allows them to survive in a relatively nutrient-deficient environment, including oxygen^28^.

T_FH_ cells in the B6.*Sle1.Sle2.Sle3* lupus mouse model show a high level of mTORC1 activation, which is associated with increased proliferation and an expansion of this T-cell subset that correlates with disease activity^8^. These autoimmune-associated phenotypes are reversed by the inhibition of glucose metabolism with 2DG. This glucose-dependence of autoreactive T_FH_ cells was not model-dependent, since it was consistently observed in three other models of spontaneous lupus^8^, induced lupus-like autoimmunity^38^, as well as in the K/BxN autoantibody-dependent model of rheumatoid arthritis^8,39^. On the other hand, the generation of pathogen-specific T_FH_ cells is not impaired by glycolysis inhibition in either lupus-prone or non-autoimmune mice^8^. This suggests that, unlike non-autoreactive T_FH_ cells requiring minimal glycolysis, autoreactive T_FH_ cells have high-glycolytic requirements, which may provide a window of opportunity for their selective elimination for reducing the production of pathogenic autoantibodies. Glutaminolysis inhibition greatly reduces immunization-induced as well as autoimmune humoral responses, as mentioned above. This includes T_FH_ cell function in both lupus-prone and non-autoimmune mice^8^. These findings indicate that, for GC B cells, glutamine is required by all T_FH_ cells (Fig. 3). T-cell-extrinsic metabolic factors contribute to T_FH_ cell expansion. Atherogenic dyslipidemia caused by a Western diet in ApoE- or LDLR-deficient lupus-prone mice increases the production of autoantibodies and the severity of lupus by expanding the number of T_FH_ cells^40^. A novel mechanism was identified by which dyslipidemia and the resulting increase in cellular cholesterol induce IL-27 production by DCs, which in turn expands T_FH_ cell responses and GC reactions to lupus-associated self-antigens^40^.

Finally, the *sanroque* mutation in the “really interesting new gene” (RING)-type ubiquitin ligase Roquin gene has shown that T_FH_ cell expansion is intimately linked to excessive GC formation, over-productive pathogenic autoantibodies such as anti-dsDNA IgG, and end-organ damage^41,42^. Interestingly, B-cell differentiation into GC B cells is limited by activated AMPK and mTOR inhibition in this Roquin mouse model of lupus^43^. A study of CD4+T-cell-specific Roquin-deficient mice revealed that Roquin-AMPK metabolic signaling is also essential for T_FH_ cell differentiation, but not for T_H_1, T_H_17, T_H_17, and Treg cells^44^. Overall, these recent studies have revealed a complex nuanced metabolic regulation of T_FH_ cells that depends on the nature of the antigen.

### T_FR_ cells

T_FR_ cells differentiate from thymic Treg cells by migrating into B-cell follicles to inhibit excessive GC responses^45^. Although T_FR_ cells share surface molecules with T_FH_ cells to localize to the GC, they also express the characteristic markers of activated Treg cells. The metabolic requirements for the differentiation and functions of Treg cells have been well studied. However, the metabolic cues that regulate T_FR_ cells remain largely unknown, since they share functional programs with both Treg cells and T_FH_ cells. IL-21 secreted by T_FH_ cells has a negative impact on T_FR_ cell differentiation through IL-21R-STAT3 signaling, leading to a heightened signaling in response to IL-2 by decreasing CD25 expression^46^. The cross-talk between the activation of the STAT and mTOR pathways has been examined relative to CD4+T-cell lineage differentiation^47^. mTOR deficiency in T cells correlates with a diminished cytokine-dependent activation of STAT4, STAT3, and STAT6, which results in a defective T_H_1, T_H_17, and T_H_2 cell differentiation, and skewing towards Treg cell differentiation^48^. In addition to its role in T_FH_ cell differentiation, mTORC1 is essential for T_FR_ cell differentiation from Treg cells and functional competence through the STAT3-TCF1-Bcl6 transcriptional axis^49^, suggesting that T_FR_ cells are metabolically more closely related to Treg cells than to T_FH_ cells. As many molecules involved in T_FR_ cell differentiation are also important in T_FR_ cell functions, alterations of the mTORC1 pathway in differentiated T_FR_ cells decrease the expression of CTLA-4, ICOS, and PD-1, which consequently leads to a decrease of T_FR_ suppressive activity^49^. Therefore, the identification of the metabolic cues that regulate Treg cell homeostasis and survival through the delicate balance between mTORC1 activation by PI3K-Akt and regulation by PTEN^50–52^, could be informative to understand how T_FR_ cells switch their metabolism in the GC. In addition, a number of metabolic cues that are important for Treg cell differentiation and function should be examined in T_FR_ cells. This includes fatty acid oxidation (FAO), which is a major energy source for Treg cells^53^, and d-mannose, which induces Treg cell differentiation by promoting activation of the latent form of TGF-β^54^. Furthermore, since HIF-1α deficiency increases the generation of Treg cells^55^ and Treg cell-specific HIF-1α-deficient mice show that glycolysis promotes Treg cell migration to inflamed sites^56^, it would be of great interest to examine the role of HIF-1α in T_FR_ cells. Indeed, the required migration of these cells to the GC hypoxic microenvironment suggests that HIF-1α may be a key determinant of T_FR_ cell metabolism.

T_FR_ cells are generally assigned to be negative regulators in the GC response. Deletion of T_FR_ cells at specific time points revealed that T_FR_ cells regulate early GC responses to control antigen-specific Abs and B-cell memory^57^. However, conflicting results have been reported regarding their role in controlling affinity maturation of B cells in response to foreign antigens and in promoting the proliferation of GC B cells through IL-10 provision^58–60^. These contrasting T_FR_ cell functions may due to change in energy sources in the GC microenvironment, or to different metabolic demands depending on the nature of the antigens, as reported for pathogen-specific and autoantigen-specific T_FH_ cells^8^. In addition, T_FR_ cells may have different origins, a canonical thymic origin^58,60,61^, as well as a naive T-cell origin that leads to antigen-specific T_FR_ cells that develop in a PD-L1-dependent manner^62^. It is possible that these two types of T_FR_ cells may be induced by different metabolic programs. Thus, a better understanding of T_FR_ cell metabolism will be crucial in dissecting the mechanisms of T_FR_ cell development and their functional role in GC responses.

### Plasma cells

One of the main goals of the GC response is to generate high-affinity-matured antibody-secreting PCs. Antibody-producing cells form a heterogeneous population that differs by their lifespan, ontogeny and anatomical location. Consequently, the metabolic requirements of PCs are also likely to differ among these various types. ATP-citrate lyase (ACLY), a rate-limiting enzyme for glucose-dependent de novo lipogenesis that produces cytosolic acetyl-CoA from mitochondrial citrate, represents a critical metabolic checkpoint for PC differentiation triggered by TLR stimulation^63^. This is a T-cell-independent, GC-independent type of humoral response in which extra-follicular short-lived PCs (SLPCs) are generated to produce germline encoded antibodies. These antibodies form immune complexes that will be retained by FDCs, which is a critical process in the incipient stage of the GC response to provide the source of selecting antigens for LZ GC B cells. As the GC response progresses, PCs secreting higher affinity antibodies show an increased lifespan to become LLPCs and localize to the bone marrow^64^. However, these LLPCs can also be generated without GC formation during T-cell-independent immune responses. LLPCs show equivalent rates of antibody expression as do SLPCs, but have a greater rate of antibody secretion^65^, suggesting that antibody secretion and PC survival are linked. However, inhibition of the mTORC1 complex in mature PCs sharply reduces antibody secretion but has no impact on survival^66^, indicating that there is no formal requirement for PCs to secrete antibodies to survive (Fig. 2).

PCs continuously utilize glucose, the main energy source during B-cell activation, once they are fully mature^67^. However, a genetic ablation of glucose transporter 1 (Glut1) still allows PC survival and antibody production, suggesting that other transporters are involved to glucose uptake^12^. Interestingly, enhanced glycolysis through hexokinase-2 overexpression favors PC differentiation rather than self-renewal, and inhibition of glycolysis leads to impaired PC differentiation or survival^68^. LLPCs predominantly use glucose for antibody glycosylation, but this glucose can be diverted towards pyruvate generation and respiration under stress conditions. LLPCs have been shown to consume high glucose and to display decreased spare respiratory capacity in comparison to SLPCs in the bone marrow^69^, thereby linking again lifespan and increased antibody secretion. PCs that lack *Tsc1*, a negative regulator of mTORC1, show an enhanced antibody secretion and ER stress response, but display shortened lifespans^70^. The same phenotypes are displayed by PCs lacking *Atg5*^71^, a gene crucial for autophagy, a process that is inhibited by mTORC1. Therefore, these data suggest that multiple metabolic mechanisms regulate differentiation, antibody secretion and survival in PCs, and that at least some of them, such as mTORC1 activation, work in opposite direction for the latter two processes.

Amino acids such as leucine and arginine activate mTORC1 in PCs, which then enhances protein synthesis^72^. LLPCs import more amino acids and have a greater capacity to import pyruvate into the mitochondria as compared to SLPCs^69,73,74^. However, the overexpression of amino acid carriers cannot compensate for the loss of the mitochondrial pyruvate carrier, Mpc2, which leads to a decreased survival and a progressive loss of LLPCs^69^. Therefore, this result indicates a unique and non-redundant role for pyruvate in LLPCs.

In contrast to LLPCs and SLPCs in the splenic and bone marrow compartments, PCs in the gut-associated lymphoid tissue accumulate glycolytic intermediates^75^. A lack of dietary vitamin B1, which is essential for promoting OXPHOS and PC differentiation, does not affect the established pool of intestinal PCs. Furthermore, treatments with inhibitors of the mitochondrial electron transport chain, such as rotenone or oligomycin, do not induce cell death in IgA^+^ PCs in vitro^75^, suggesting that intestinal PCs may be able to survive entirely on ATP derived from glycolysis. Since intestinal IgA^+^ PCs utilize short-chain fatty acids as a carbon source in the electron transport chain^76^, dietary palmitic acid increases IgA production and the number of IgA^+^ PCs via the production of S1P and cellular proliferation^75^. Stable-isotope tracing studies have also implicated glutamine uptake in PC function, suggesting that glutamine is used for oxidation, and for the synthesis of other amino acids and as well for antibody protein synthesis^77^. Thus, the same nutrients required to synthesize antibodies, such as amino acids, are also utilized for PC energy and survival. However, PC metabolism may be adjusted to their environment and their respective functions.

Finally, PC metabolism has been largely studied in experiments in which differentiation and survival are coupled to cell activation. Consequently, it is difficult to assess whether the effects observed with metabolic inhibitors affect B-cell activation, proliferation of activated B cells, or PC differentiation or survival^67,74^. Only a few studies have investigated the metabolism of memory B cells. ATG7-mediated autophagy is necessary for the survival of memory B cells^78,79^. Furthermore, glycolysis and mTORC1 activation were observed in in vitro differentiation into plasmablasts from human memory B cells via TLR ligands and IFNα^80^. It is not clear, however, whether it was critical to these metabolic requirements that the starting material was memory instead of naive B cells. Therefore, more detailed studies are needed in which metabolic regulators are targeted specifically to plasmablasts, PCs, and memory B cells to separately assess their effect on survival and function.

## Conclusion

While we and other groups in the field of GC biology have defined some of the specific metabolic pathways of the GC response, these recent studies have highlighted the complex relationships that exist between metabolic networks and their consequences on immune activation during the GC response. GC are transient structures located within B-cell follicles during an immune response, which means that all GC lymphocytes require the processes of cell activation, migration, maturation, and selection. In these complex and tightly regulated processes in the GC, cells demand different carbon energy sources and metabolic pathways depending on their developmental stage. An additional complicating factor to a better understanding of the metabolic requirements of GC reactions is the inter-dependence of T_FH_ and GC B cells, and the limited tools that can be used to selective target each of these populations.

We have shown that nutrient demand and metabolic pathways are not the same in T_FH_ cells driven by different antigens, suggesting that GC originating from different pathogens may have different metabolic demands for optimizing their responses. Currently, distinct T_FH_ cell subsets, T_FH_1, T_FH_2, and T_FH_13 cells, are characterized by the nature of the inducing pathogens that also drives the differentiation of the corresponding helper CD4^+^ T-cell subsets^81^. These cells require different activation signals and transcriptional consequences. More importantly, these distinct T_FH_ cell subsets dictate the outcome of the most appropriate antibody response. Thus, it will be of great interest to profile the metabolic pathways in each GC cell type under the different type of pathogens. In addition, the metabolism of non-lymphocyte players such as the DCs that initiate T_FH_ cell differentiation, FDCs and tingible body macrophages, should also be considered, although technical issues are still a limitation for the analysis of these populations. A full metabolic picture of the GC could guide the design of better vaccines and provide therapeutic approaches for antibody-mediated autoimmune diseases.
